# Incorporating concepts of inequality and inequity into health benefits analysis

**DOI:** 10.1186/1475-9276-5-2

**Published:** 2006-03-28

**Authors:** Jonathan I Levy, Susan M Chemerynski, Jessica L Tuchmann

**Affiliations:** 1 Exposure, Epidemiology and Risk Program, Department of Environmental Health, Harvard School of Public Health, Landmark Center Room 404K, P.O. Box 15677, Boston, MA, 02215, USA; 2 Department of Environmental Health Sciences, Johns Hopkins Bloomberg School of Public Health, 615 N. Wolfe St., Baltimore, MD 21205, USA

## Abstract

**Background:**

Although environmental policy decisions are often based in part on both risk assessment information and environmental justice concerns, formalized approaches for addressing inequality or inequity when estimating the health benefits of pollution control have been lacking. Inequality indicators that fulfill basic axioms and agree with relevant definitions and concepts in health benefits analysis and environmental justice analysis can allow for quantitative examination of efficiency-equality tradeoffs in pollution control policies.

**Methods:**

To develop appropriate inequality indicators for health benefits analysis, we provide relevant definitions from the fields of risk assessment and environmental justice and consider the implications. We evaluate axioms proposed in past studies of inequality indicators and develop additional axioms relevant to this context. We survey the literature on previous applications of inequality indicators and evaluate five candidate indicators in reference to our proposed axioms. We present an illustrative pollution control example to determine whether our selected indicators provide interpretable information.

**Results and Conclusions:**

We conclude that an inequality indicator for health benefits analysis should not decrease when risk is transferred from a low-risk to high-risk person, and that it should decrease when risk is transferred from a high-risk to low-risk person (Pigou-Dalton transfer principle), and that it should be able to have total inequality divided into its constituent parts (subgroup decomposability). We additionally propose that an ideal indicator should avoid value judgments about the relative importance of transfers at different percentiles of the risk distribution, incorporate health risk with evidence about differential susceptibility, include baseline distributions of risk, use appropriate geographic resolution and scope, and consider multiple competing policy alternatives. Given these criteria, we select the Atkinson index as the single indicator most appropriate for health benefits analysis, with other indicators useful for sensitivity analysis. Our illustrative pollution control example demonstrates how these indices can help a policy maker determine control strategies that are dominated from an efficiency and equality standpoint, those that are dominated for some but not all societal viewpoints on inequality averseness, and those that are on the optimal efficiency-equality frontier, allowing for more informed pollution control policies.

## Background

Environmental policy decisions in the United States are often informed by two lines of evidence. Risk assessment techniques can quantify the magnitude of public health benefits anticipated from proposed control strategies (hereafter referred to as health benefits analysis), a process required for all regulations with significant economic impacts [1]. As these analyses are conducted within the context of benefit-cost analysis, the focus has historically been on aggregate benefits as a measure of the "efficiency" of the control measure, with little consideration of either the baseline distribution of risk or how the benefits are distributed across the population.

At the same time, questions about environmental justice and the distribution of affected populations are influential in many environmental policy decisions. An Executive Order requires that all federal agencies identify and address disproportionate impacts of policies and programs on minority and low-income populations [2]. Given this Executive Order, along with growing interest among community members and researchers, environmental justice concerns dominate how many communities view environmental issues [3,4].

Although many policy decisions are influenced by both mandates, there has been little systematic consideration to date of how concepts relevant to environmental justice could be incorporated into the health benefits analysis framework (or, more generally, how health benefits analysis could better incorporate variability in risk). Some environmental justice analyses provide quantitative estimates or indicators of inequality, but rather than quantifying health risks, focus on issues such as demographic patterns of populations proximate to hazardous waste sites [5-8] or sources of toxic chemicals [9-11]. This can help determine if facility siting was discriminatory but may have little direct relevance to the distribution of health outcomes. Many risk-based equity studies stop short of quantifying health benefits, instead characterizing inequities in air toxic emissions [12-14] or concentrations [15], or risk scenarios [16,17]. A focus on concentrations or exposures is often logical, given a lack of evidence on health outcomes, but potentially omits important concepts of susceptibility or non-linearity in dose-response functions. Even those studies that applied inequality indicators in risk assessment [18,19] did not address issues related to pollution control (rather than the baseline distribution of risk) or the implications of choosing one indicator over another.

Although past studies have not explicitly brought concepts important to environmental justice and risk assessment together, there is growing interest and improved methods to do so. For example, cumulative risk assessment [20] quantitatively addresses some concerns of environmental justice advocates by focusing on community and individual risks, rather than specific contaminants. Increasing scientific information about socioeconomically patterned exposure heterogeneity or susceptibility linked to environmental contaminants [21] can be used to better estimate the distribution of health benefits. In general, multiple studies have articulated approaches for incorporating distributional concerns into economic cost-benefit analysis [22,23], albeit without the risk assessment components. Thus, the development of methods to better articulate inequality issues within health benefits analysis would be timely.

The critical question is therefore: can one appropriately quantify inequality or inequity in health benefits analysis, and if so, how? Extensive work has been conducted to date on the development and evaluation of indicators of inequality in other contexts [24-26], and it is not our intent to replicate this work. Rather, we focus on developing the most appropriate method for determining whether a pollution control strategy increases or decreases health inequalities.

To achieve this goal, we first present key definitions needed to understand the similarities and differences in the frameworks of health benefits analysis and environmental justice analysis. We propose axioms that an inequality indicator for health benefits analysis must follow, including both general axioms previously considered and specific axioms in the context of health benefits analysis. We use these axioms to evaluate indicators developed or applied in previous studies and illustrate the potential interpretability of selected indicators through an illustrative pollution control example in which efficiency-equality tradeoff frontiers are developed. We conclude by discussing strengths and weaknesses of our proposed approach and by outlining areas in which further methodological development is required.

## Definitions and concepts

In this section, we present key concepts underlying both health benefits analysis and environmental justice analysis. We focus on developing working definitions of equality and equity, highlighting the differences in how environmental justice and risk assessment/health benefits analysis understand these concepts. A deeper understanding of equality and equity also clarifies the extent of our ability to quantify such measurements.

### Definitions: Health benefits analysis

Health benefits analysis involves the application of risk assessment techniques to estimate the magnitude of health benefits associated with pollution control measures. These measurements are then used as a component of benefit-cost analysis, which involves estimation of the incremental costs and benefits associated with a given policy, often both expressed in monetary terms for comparability. While health benefits analysis can consider distributional information, the aggregate societal benefit has been the measure of concern in most applications to date. More refined distributional information has generally not been utilized except to the extent that it improves the aggregate benefit estimates. This should be differentiated from other risk assessment applications, where the objective may be to determine whether a maximally exposed individual has a risk exceeding a predefined threshold.

Conventionally, a distinction is made between risk assessment, the use of the factual base to define the health effects of exposures of individuals to hazardous materials, and risk management, the process of selecting among policy alternatives by balancing risk assessment findings with social, economic, and political concerns [27]. Multiple studies have debated whether this careful distinction leads to more objective risk assessment or imposes its own set of value judgments that impair decision-making [28,29]. Whether or not the differentiation between risk assessment and risk management is useful and meaningful, it reflects a desire to ensure that subjective judgments within the risk assessment are made explicit, and that important choices that may influence policy decisions are not hidden from the policy maker.

### Definitions: Environmental justice analysis

While it is problematic that health benefits analysis has not historically incorporated distributional impacts, linking environmental justice with health benefits analysis is further complicated by difficulties in developing functional definitions of justice, equity, or equality. These definitions would be needed either for formal analyses or for comparisons across studies. We provide basic definitions of equality and equity, and consider how inequality could be formally aggregated, before drawing conclusions about the implications for health benefits analysis.

Equality is one of the core components of justice, characterized by homogeneity or sameness among individuals or social groups [30]. It is often depicted as uniformity in rights or experiences despite differences in resources, capabilities and backgrounds. Equality is a fundamentally comparative concept, judged on the basis of relative factors that constitute an individual's or group's experience within society, such as income, utility, resources or capacity [31]. Equality can be understood along a continuum from origin to procedure to outcome, with numerous categorizations including equality of opportunity, freedom from discrimination, equal treatment before the law, equal benefit, equal status and equality of results. Within the context of health benefits analysis, despite inherent differences among individuals and initial unequal conditions [32], inequality can decrease through our ability to alter regulations and reduce pollutant exposures and/or health risks. Of note, equality could theoretically be improved by "leveling down" and increasing health risks among low-risk individuals, but this would most likely not be sensible in a context where efficiency and aggregate social welfare were also of interest. Explicit development of efficiency-equality tradeoff frontiers for pollution control policies can empirically test these preferences.

In contrast, the concept of inequity identifies the subset of inequalities that are deemed unjust and unfair by a socially-derived calculus [33]. The three central determinants of inequity are whether the difference is avoidable, undeserved, and remediable [34]. For example, health inequalities stemming from genetic differences or freely-chosen health damaging behavior would not be considered inequitable; variations resulting from unknown exposure to unhealthy working conditions or limited social mobility would be categorized as inequitable [33]. Equity is divided into a number of subcategories. One common division is between procedural and outcome (or distributive) components. Procedural equity focuses on fairness in the processes that gives rise to outcomes [35]. Examples include inclusive and open participation in public proceedings and making specific attempts to include affected or disadvantaged individuals in the debate. Outcome equity imposes justice on the ultimate distribution of resources at the completion of the process, requiring the final outcome – but not the intermediate process –to meet the test of fairness.

These definitions make it clear that quantitative comparisons of inequality will be more feasible than quantitative comparisons of equity or justice. This leads to the question of how one can formally compare inequality claims. One significant question involves whether the social unit of concern would be the individual or social groups. Since important interindividual inequality can be masked by group averages [36,37], individual-level information is ideal, but may not be practical or meaningful in some contexts. A determination regarding the reference point for evaluating claims must be considered next. Temkin provides four useful conceptualizations, depicted in Table 1, which can be used to determine the basis for comparison when judging inequality [36]. A third component of comparing inequality claims involves aggregating claims and assessing their relative importance. Three methods typically used for calculating the extent of a given inequality are summarized in Table 2[36].

**Table 1:** Four Approaches for Inequality Comparisons [36]

**Concept**	**Who/what is reference point?**	**Benefits**	**Limitations**
Relative to average	The mean inequality of all individuals within a group	Often the easiest metric to obtain and compare. Can be used for both individual and group vs. group comparisons.	Group averages can mask important inter- individual inequalities
Relative to the best- off	Experience of the single best-off person in society	Can identify differences between poorest and richest individuals; easy to quantify for income	The best-off may not be a realistic equality standard, and the experience of the best off person may be difficult to quantify in a risk context
Relative to all those better off	The range of experiences of all those who are better- off than a given person/group	Allows a deeper understanding of scope of inequality within a group	Hard to identify the level at which claims would be deemed unequal
Relative to the best- off person whose condition is not anomalous	Compares individual claims to a determined "good enough" level	Allows for a more reasonable expectation of equality	Hard to define "not anomalous" in real-world context

**Table 2:** Methods of Devising Composite Inequality Measures [36]

**Model**	**Method of Calculation**
Additive principle	Sum of each individual complaint of inequality; the higher the number, the greater the inequality
Weighted additive principle	Claims with greater significance are given greater value in the assessment
Maximim principle	The extent to which societal institutions maximize the average level of the worst-off group

### Conclusions: Differences in definitions and frameworks

Given the above definitions, there are clear distinctions in how environmental justice and health benefits analyses would define and incorporate notions of equality and equity. First, environmental justice and health benefits analysis focus on different points on the continuum from origin to procedures to outcome. Environmental justice advocates often focus on procedural fairness, with the belief that inequities in health outcomes stem from the lack of influence of low-income or minority communities on facility siting decisions and institutionalized discrimination in the application of environmental laws [38]. The objective is to improve outcomes, but with a focus on process equity as a means to achieve this end.

On the other hand, health benefits analysis is explicitly focused on quantifying end states (exposures and health outcomes) as a means of affecting public policy, without regard to procedural issues or community involvement or input [39]. Although this difference in focus is important, environmental justice advocates are ultimately concerned about outcomes. Thus, techniques that can appropriately quantify the magnitude and distribution of exposures and health risks should be relevant.

Additional differences would be related to how quantitative comparisons of inequality are made. Most environmental justice analyses compare demographic patterns at varying proximities to pollution sources [6,7] or exposures across defined socioeconomic or racial groups [15], clearly involving group-level comparisons. While health benefits analysis is generally conducted at geographically aggregated levels due to technical or financial constraints in accurately assessing an individual's experience, the "gold standard" would involve the use of individual-level information when available.

In addition, environmental justice advocates typically compare the inequality claims of disadvantaged groups to experiences of more privileged geographic and demographic groups, implicitly employing the "relative to all those better off" comparison (Table 1). This is not a precise fit, given the focus on group experience rather than that of individuals and the fact that the reference group for environmental injustice claims may change over time or across policies. For health benefits analyses, comparisons other than "relative to average" or "relative to all those better off" may often be impractical due to data limitations and the difficulty of characterizing risk at the tails of the distribution.

Finally, the methods for aggregating individual claims may differ (Table 2). Environmental justice advocates openly incorporate weighting and a maximin approach into their analyses in order to fully consider socioeconomic factors; as above, these measures often vary by situation. The strictly additive nature of health benefits analysis (as conventionally applied) may run counter to this more adaptable approach.

These differences in frameworks are all based on concepts of inequality, and do not address whether inequality or inequity would be more appropriate. Equity, unlike equality, does not lend itself to being quantified, since it incorporates elements of socially constructed fairness and justice that are beyond the scope of mathematical analysis. Since questions of societal fairness (and therefore equity) are central in environmental justice, and given the above issues, it would seem that the tensions in orientation between environmental justice and quantitative health benefits analysis are impossible to reconcile. That being said, explicit incorporation of exposure heterogeneity and susceptibility information can allow us to better evaluate equality in health benefits analysis, which can partially inform determinations of equity if relevant individual information is included. We therefore aim to develop quantitative indicators of inequality for health benefits analysis that have the flexibility to incorporate sufficient information to inform outcome equity judgments for environmental justice advocates.

In summary, our definitions emphasize that health benefits analysis provides a measure of the efficiency of a control strategy, aggregated across the population. While this framework has not typically incorporated inequality, a quantitative measure of inequality that takes into account the above definitions is feasible and interpretable. Ultimately, this would allow one to evaluate the impact of pollution control policies on both efficiency and equality, and determine which policies are dominated from both perspectives and which lie on an optimal tradeoff frontier.

## Results

### Axioms for inequality indicators for health benefits analysis

In this section, we review the axioms proposed in past evaluations of inequality indicators, evaluating each for its relevance to health benefits analysis. We then propose a set of additional axioms that should be imposed for an inequality indicator in this context to be meaningful and interpretable.

#### 1. Pigou-Dalton transfer principle

This axiom (as applied to income inequality) requires that an indicator not decrease when income is transferred from a poorer to a richer person, and that it decrease when income is transferred from a richer to a poorer person [40]. This is predicated on the mean of the distribution remaining unchanged and the amount of the transfer being less than the existing gap [36,40]. This axiom considers inequality as a gradient rather than a threshold concept; in other words, even a transfer from the wealthiest to the second-wealthiest person in society should reduce inequality. The application for health benefits analysis is analogous: if a policy results in the redistribution of risk from high-risk to low-risk populations, then an inequality indicator should decrease accordingly.

The Pigou-Dalton transfer principle has not been seriously disputed in the income inequality literature, given its importance for intuitive interpretation of inequality indicators. While some have argued that it is less interpretable for health given the difficulty of "transferring health" [41], pollution control decisions implicitly involve choices about redistribution of exposures and therefore health. Given this, and since our objective in interpreting changes in risk distributions parallels interest in income inequality, we accept this axiom for health benefits analysis. The principal difference relative to income inequality assessment is empirical rather than theoretical; some indicators violate the Pigou-Dalton transfer principle under circumstances that are relatively unimportant for income inequality but may be more significant for risk inequality (i.e., transfers among individuals well above the mean).

#### 2. Scale invariance

Scale invariance requires that an indicator not change given uniform proportional changes across the population. This would imply that relative comparisons are most important for inequality, as opposed to translation invariance (where an indicator would not change given uniform additive changes across the population), which would focus on absolute differences. While it is clear that this axiom should be followed for the case of changing income to different currencies [24], there has been extensive debate about whether scale invariance for real changes in income would or would not be desirable. Some have argued that proportional increases in income should increase inequality, as the absolute gap between the poor and wealthy has increased [42], and the assets added to society have not been distributed in an equitable fashion [36]. Others have argued that proportional increases in income should decrease inequality, given the diminishing marginal utilities of income [43]. Of course, many accept scale invariance as a useful and important axiom as well.

For health benefits analysis, the marginal utility of risk reduction is likely not constant across levels of health risk. It could be argued that this uneven marginal utility supports explicitly rejecting scale invariance, especially since the question of unit conversion does not pertain (as it would with income) unless intermediate measures such as exposure (which is based on concentration units that could be converted) are used. On the other hand, relative differences in health risk could be more meaningful than absolute differences [24], especially given that risk calculations are generally multiplicative with uncertain relative risks associated with exposures. Survey evidence suggests that the degree to which scale and/or translation invariance is accepted depends on the baseline income/risk and the magnitude of the increment [44], and that there is significant support for intermediate positions [45].

For this axiom, since there are compelling arguments in either direction, we conclude that either scale invariant or non-scale invariant indicators may be appropriate, with the choice potentially dependent on the context. For problems involving small changes in a risk that does not vary substantially across the population, the marginal utilities of risk reductions will be reasonably similar and scale invariance may be more appropriate.

#### 3. Anonymity

This axiom requires that an inequality measure be independent of any characteristics of individuals other than the welfare indicator whose distribution is being evaluated [26]. This axiom has been disputed, with some arguing that evaluation of inequalities without consideration of population characteristics is inappropriate given the importance of social factors as predictors of health and well-being [46]. Anonymity has some appeal, as it avoids identifiability and situations where the indicator can change without health risk changing. However, environmental justice by definition is concerned with sociodemographic factors and comparisons between groups, and linking inequality with inequity requires such information. Moreover, understanding geographic patterns of health risks may facilitate the development of pollution control strategies. Thus, although there are some contexts in which anonymity may be reasonable, we prefer indicators where relevant individual characteristics can be incorporated.

#### 4. Subgroup decomposability

An indicator that is subgroup decomposable (or additive separable) can have total inequality divided into constituent parts of the distribution. Typical applications might include segmentation of inequality into within-group versus between-group inequality, or evaluation of the contribution of various population subgroups to overall inequality [26].

Some have argued that for inequality, the whole may not be identical to the sum of the parts [36], since interpretation of inequality in a subpopulation may depend on what is occurring in the rest of the population. However, the practical appeal of a subgroup decomposable measure for health benefits analysis is substantial. It enhances interpretability and allows population characteristics (such as socioeconomic status) to be incorporated into the analysis, with consideration of whether societal inequality is largely explained by between-group differences. Most importantly, a subgroup decomposable measure can incorporate key concepts from risk assessment (i.e., subpopulation susceptibility) with concerns from environmental justice (i.e., distribution of impacts across population subgroups of concern).

#### Additional axioms

Other standard axioms, such as analytic tractability (the indicator should be computable in standard applications), appropriateness (the indicator should adequately reflect perceptions about inequality), and normalization (the indicator should either follow a defined range or be able to be transformed to do so) [24-26], are fairly uncontroversial in this context or others and do not require further attention. Others may be controversial in other settings but are largely irrelevant to health benefits analysis. The principle of population (invariance of an indicator to the replication of the population) is one example, since the population is fixed across control policy options in most health benefits analyses.

In addition to these conventional axioms, for an indicator of inequality to be meaningful for health benefits analysis, it must fulfill additional criteria. As mentioned above, quantification of health benefits contains multiple assumptions and uncertainties not found in evaluations of income inequality. While the additional axioms pertain more to the welfare measure than the indicator itself, some are generalizable to other contexts.

#### 5. The analyst must not impose a value judgment about the relative importance of transfers at different percentiles of the risk distribution

As mentioned above, risk assessment has often been distinguished from risk management, partly to reduce subjectivity within risk assessments, or, at a minimum, to ensure that any subjective judgments made within the risk assessment are presented explicitly. When quantifying inequality in health benefits analysis, any single indicator will involve implicit choices about the relative importance of transfers at different percentiles of the distribution, whether the relevant comparison is with the average or the best-off individual, and so forth. Even an indicator with equal weights across the population involves an implicit value judgment. By choosing a single way of quantifying inequality, the analyst has imposed his/her judgment about these issues.

This problem can be solved one of two ways. First, the analyst could use an indicator that includes an explicit parameter that changes the weights placed on various percentiles of the distribution. In that way, a single weighting scheme would not be endorsed, as the sensitivity of policy decisions to values of this parameter should be analyzed. Alternatively, the analyst could use multiple indicators that reasonably cover most relevant viewpoints about inequality, even if the individual indicators do not allow for multiple weighting schemes. Ideally, both approaches would be used. In any case, the sensitivity of the indicator to transfers in various portions of the distribution should be explicitly presented, so that the decision maker correctly interprets the outputs of the analysis.

#### 6. The welfare measure must be as close to a measure of health risk as possible. If quantifying risk is impossible or there is no differential susceptibility, then exposure should be evaluated. If quantifying exposure is impossible or there is no differential exposure, then concentrations in relevant media should be evaluated

Since our objective is to inform health benefits analyses, it is important to base the inequality indicator on health evidence. Although quantitative evidence of differential susceptibility of population subgroups to a pollutant is often limited, this axiom emphasizes the importance of this evidence. At a minimum, the analyst should attempt to determine if there are any differences in baseline health status that would influence the distribution of health impacts. In other words, if a pollutant increases everyone's risk of an emergency room visit for asthma by 5%, then individuals or groups with a higher baseline rate of asthma emergency room visits (such as low-income African-Americans) would contribute a disproportionate amount to the total public health burden [47]. Differential susceptibility from a relative and absolute perspective should be explored.

Since there are often multiple health outcomes associated with a single exposure, the welfare measure should be based when possible on aggregate severity-weighted health outcomes (such as quality-adjusted life years lost). If evidence is lacking to estimate these measures, or if use of an aggregate health measure would result in the inability to reasonably incorporate susceptibility, a single health outcome should be chosen that either contributes most to severity-weighted health measures or that is of interest to decision makers for other reasons. Given sufficient evidence, the geographic and demographic distribution of risk per unit exposure will differ by health outcome, since susceptibility is disease-specific. If multiple health outcomes are of concern but cannot be aggregated, the inequality indicator should be calculated for each, and the ranking of the policy options should be compared.

#### 7. The inequality indicator should not be applied without consideration of the baseline distribution of risk

This axiom is intended to capture the concern regarding cumulative environmental burdens placed on low-income or minority communities, which might otherwise be omitted from the analysis. This issue is especially important if a pollutant has a non-linear dose-response function, where knowing the current level of exposure is critical in quantifying the incremental health risk. This would generally involve estimation of baseline exposures to the pollutant of concern and the baseline incidence of the health outcome in question, including at small geographic scales and across sociodemographic variables of interest. A more refined approach in a multipollutant context would adopt a cumulative risk assessment framework [20], systematically assessing stressors with similar mechanisms of action and exploring the possibility of synergistic or antagonistic effects with the pollutant(s) of interest for the analysis.

#### 8. The inequality indicator should be estimated for the geographic scope and resolution that are used for the health benefits analysis, but the sensitivity of the findings to scope and resolution should be evaluated. In particular, an inequality indicator should be estimated with the finest geographic resolution possible, given available data and analytical capabilities

As mentioned earlier, individual-level information is the theoretical ideal when making determinations of inequality, but data and analytical limitations make coarser resolution necessary. Health benefits analyses often have county-level resolution or greater [48,49], limited by the complexity of atmospheric dispersion models and by epidemiological evidence based on air pollution measurements at central site monitors. This leads to obvious difficulties, since multiple studies have shown that the findings of an equity analysis (as expressed by demographic patterns near sources) depend on the geographic resolution of the analysis [6,14,50,51]. Larger geographic aggregates can contribute to ecological fallacies with the loss of higher-resolution data, unless the populations are homogeneous with respect to all characteristics of interest.

Whereas there is a theoretically correct geographic resolution for an inequality analysis, the appropriate geographic scope of the analysis is harder to define. Health benefits analyses are often based on political boundaries (state, regional, or national scope) rather than boundaries based on exposure concepts. The inequality assessment should use the scope selected for the health benefits analysis for comparability, but sensitivity analyses should be conducted to determine if the optimal policies are stable with respect to modifications to the geographic scope.

#### 9. When efficiency-equality tradeoffs are important for policy decisions, the inequality indicator should be derived for multiple competing policy alternatives. If this is not possible, qualitative interpretations are most appropriate

One of the stipulations related to the Executive Order requiring health benefits analyses for major regulations [1] was that the agency must consider a number of regulatory alternatives and select the least costly, most cost-effective, or least burdensome alternative to achieve the stated objectives [52]. In general, benefit-cost analyses or cost-effectiveness analyses are most useful when a baseline/reference case is developed and multiple interventions are compared against that baseline/reference case.

In our case, it is even more important to consider multiple control options. An inequality measure may be sensitive to geographic scale, geographic resolution, pollutant, and health outcome, among other factors. Unlike premature deaths or dollars spent, inequality indicators are difficult to compare across applications. If a decision maker were told how much an inequality indicator changed as a result of a policy change, along with an estimate of health benefits, it would be difficult to use this information by itself to make definitive policy decisions (aside from the qualitative determination of whether inequality increased or decreased). Evaluating how the inequality indicator changes as a result of multiple potential alternatives allows for the determination of efficiency-equality tradeoffs (if any). Aggregate health benefits and changes in inequality can be plotted against one another to determine which policies are dominated from both efficiency and equality standpoints, and which are on an optimal trade-off frontier.

#### Summary

In summary, an indicator of inequality for health benefits analysis must follow a set of rules in order to be meaningful and interpretable. Of the standard axioms, the Pigou-Dalton transfer principle should not be violated, while subgroup decomposability adds tremendously to interpretability and would therefore be useful in most environmental justice applications. We have proposed five additional axioms that are specific to health benefits analysis. While there are undoubtedly many additional axioms that could be imposed, this subset should provide an interpretable and meaningful inequality indicator without being excessively constraining. In the next section, we review some key inequality indicators and interpret them given the above axioms.

### Evaluation of Key Inequality Indicators

To select an appropriate inequality indicator for health benefits analysis, we first surveyed the literature to find measures previously applied or proposed in the economic, sociologic, or environmental science fields. Measures were collected through searches in Social Sciences Citation Index, Science Citation Index, Medline (Ovid) and PubMed in June and July 2004, using the keywords: inequality and health, inequality measure/measurement/indicator, equity measure/measurement/indicator, and all specific inequality measure names mentioned elsewhere. The intent was not to provide an exhaustive list of all publications, but to find a representative sample of indicators.

Since any inequality indicator will implicitly or explicitly incorporate social judgments and will capture an underlying philosophy about inequality [24], it was important to describe and understand the characteristics of each indicator across a number of dimensions. Thus, for each inequality measure, we compiled information related to its quantitative definition, original derivation and rationale, underlying philosophy, and past applications. We then evaluated each measure relative to the axioms proposed above. Of note, axioms 6–9 are related to the context in which the indicator is used, so we focus herein on the Pigou-Dalton transfer principle, subgroup decomposability, and the ability to avoid imposing a value judgment about the relative importance of transfers at different percentiles of the risk distribution.

We examined two major categories of inequality measures that have some important contrasts – summary measures and social welfare functions. Summary measures (or positive measures) function to create an absolute measure that matches the degree of inequality inherent, but do not explicitly incorporate a concept of social welfare [43]. In contrast, social welfare functions (or normative measures) are derived for the explicit formulation of social welfare, which some have argued is preferable to summary measures [24]. Normative measures lack some of the implicit assumptions regarding the form of the social welfare function, which may limit the comparability of the measures but can also be a primary attribute and strength of these measures.

Although many indicators have previously been proposed or applied, we found 19 distinct indicators through our search that merited further investigation. We focus our discussion on five measures (two summary measures and three social welfare functions) that were either commonly used in past inequality applications, were discussed at length in review articles, or were likely candidates given our axiomatic approach.

#### 1. Gini index

The Gini index was the most commonly used inequality measure, with applications that were predominantly based on income [53-60] but also included health [61-63], and environmental health risk [16,18] or emissions [12,13]. It is formally defined as one-half the relative mean difference, which is the arithmetic average of the absolute differences between pairs of measures (Table 3). It can also be derived from the Lorenz curve, equaling twice the area between this curve and the 45-degree line representing total equality [54]. The Gini coefficient reflects the measurement of individual complaints relative to all those better off, using an additive principle of equality [36].

**Table 3:** Summary of Inequality Indicators Evaluated for Health Benefits Analysis.

	**Gini index**	**Variance of logarithms**	**Squared coefficient of variation**	**Atkinson index**	**Mean log deviation**	**Theil's entropy index**
Formula		Where z_i _= ln(x_i_)		where ε = inequality aversion (range from 0 to infinity)		
Approach for comparisons	Relative to all those better off	Relative to the average	Relative to the average	Relative to the average	Relative to the average	Relative to the average
Method for aggregation	Additive	Weighted additive	Weighted additive	Weighted additive	Weighted additive	Weighted additive
Principle of transfers?	Y	N (fails for transfers at high levels)	N (fails for transfers at high levels)	Y	Y	Y
Subgroup decomposable in standard form?	N (unless subgroups strictly ordered by income)	N	Y (within-group and between-group not independent)	Y (although not strictly additive)	Y	Y
Avoids value judgment about weights?	N (in standard application; extended Gini can address)	N	N	Y	N	N
Conclusions	Rejected as stand- alone indicator; potentially useful for sensitivity analyses	Rejected	Rejected	Accepted	Rejected as stand-alone indicator; useful in combination with other indicators	Rejected as stand-alone indicator; useful in combination with other indicators

The Gini index satisfies the Pigou-Dalton transfer principle, but has a number of limitations in its standard form. The transfer sensitivity depends on the rank of incomes rather than the absolute values. This yields weights that appear somewhat arbitrary [64], with weights that are translation invariant and with more weight attached to transfers in the middle of the distribution [54,65]. Moreover, the Gini index is not subgroup decomposable into within-group and between-group components unless subgroups can be strictly ordered by income [55,65-67]. Finally, it imposes an implicit value judgment about the segments of the distribution that matter most. Extensions to the Gini have been developed that can incorporate varying value judgments [59,66], enhancing its utility for health benefits analysis. However, given the difficulty with subgroup decomposability and the fact that the weights attached to health risks would not change with a constant addition of risk, the Gini index may not be interpretable for many combined environmental justice/health benefits analyses, but could be considered in sensitivity analyses.

#### 2. Variance of logarithms

The variance of logarithms is a straightforward measure of dispersion, which has previously been considered in reviews of inequality indicators [24,54]. It has the theoretical appeal of simplicity, especially for lognormally distributed data (Table 3). Like a number of simple measures of dispersion, the variance of logarithms represents a relative to the average view of individual complaints and a weighted additive principle for summation across individual complaints [36].

However, the variance of logarithms violates the principle of transfers, with marginal transfers from high-risk to low-risk increasing the variance of logarithms if the high value is greater than e times the geometric mean of the distribution [68]. Moreover, it implicitly attaches more weight to transfers at the low end of the distribution than to transfers at the high end of the distribution [36]. The variance of logarithms is only decomposable if geometric means replace arithmetic means in the subgroup data, which places greater emphasis on the low end of the distribution [69]. The variance of logarithms is therefore not applicable to health benefits analysis.

#### 3. Squared coefficient of variation (SQV)

The summary measures considered above are not ideal for health benefits analysis, in part because of difficulties with subgroup decomposability. The family of generalized entropy measures was derived specifically to be decomposable, allowing for neat decomposition into within-group and between-group terms, in which total inequality is an additive function of between-group and within-group inequality [65,69]. Moreover, these generalized entropy measures incorporate a constant, θ, which determines the relative sensitivity of the entropy indices [69]. When θ is less than 1, the index is more sensitive to the lower end of the distribution, and when θ is greater than 1, there is more sensitivity at the high end. Generalized entropy measures therefore have significant appeal for health benefits analysis.

The first of the generalized entropy measures we consider is the squared coefficient of variation (SQV; Table 3). This represents the generalized entropy measure when θ equals 2. As in any case where θ is specified, the benefit of having the sensitivity parameter is lost unless multiple generalized entropy measures are used in the analysis. The SQV, or the coefficient of variation itself, has been used in past economic applications [54,55,70]. Although the SQV is decomposable, the within-group components and their weights are not independent of the between-group components [71]. In addition, as with the variance of logarithms, the SQV fails the principle of transfers for high values. Thus, we reject the SQV for health benefits analysis.

#### 4. Atkinson index

The Atkinson index is a member of the generalized entropy family of indicators, explicitly incorporating normative judgments about social welfare [54,66,72]. The Atkinson index was derived for income inequality [54] and has been applied in this context [53,55,57,61,73,74] as well as for health [75] and access to health care [76].

For income inequality, it is derived by calculating the equity-sensitive average income, which is defined as the level of per capita income which, if uniformly possessed, would make total welfare exactly equal to the total welfare generated by the actual income distribution. Societal preferences for equality are incorporated through an explicit parameter ε, which is ordinally equivalent to 1-θ in the generalized entropy equation for values of θ < 1 [26] (Table 3). When ε > 0, there is a societal preference for equality, and as ε rises, society attaches more weight to income transfers at the lower end of the distribution. In an extreme case, an infinite value of ε would represent the Rawlsian concept of providing the greatest benefit to the least advantaged [36,77]. Typical values of ε applied in the literature range from 0.25 to 2.0 [54,57,61].

Aside from including an explicit parameter for degree of inequality averseness, the Atkinson index fulfills the Pigou-Dalton transfer principle and is subgroup decomposable. Given the non-linear transformation of the generalized entropy index, the between-group and within-group components do not sum exactly to total inequality [65]. Instead, the functional relationship can be expressed as:

(1 - A_total_) = (1 - A_within_)(1 - A_between_) [78].

Thus, the Atkinson index satisfies our proposed axioms and merits consideration.

#### 5. Theil's measures of inequality

The final generalized entropy measures we consider are two indices derived by Theil [71], the mean log deviation (for which θ = 0) and the Theil's entropy index (for which θ = 1) (Table 3). Both the mean log deviation [57,70] and the Theil's entropy index [53,55,61] have had multiple applications in the economic literature. The two Theil inequality indices are additively decomposable with independence of within-group and between-group inequality [69,70], offering different weighting options for the subgroups, and satisfy the Pigou-Dalton transfer principle. Moreover, the indices have some theoretical appeal based upon concepts from information theory – the expected information content of a situation can be considered as the sum of the information content weighted by the respective probabilities. However, these indices have been criticized for the lack of intuition regarding the calculation of the "average of the logarithms of the reciprocals of income shares weighted by income shares" [43], and the upper bound of the Theil's entropy index depends on sample size. Since neither of these critiques invalidates the use of Theil's indices, we do not reject these indices, although the specification of θ implies that they should only be used in combination with other indicators, most likely in sensitivity analyses.

#### Summary

We conclude that the Atkinson index is the indicator that best addresses the needs of inequality assessment in health benefits analysis, as it does not violate the Pigou-Dalton transfer principle, allows for subgroup decomposability, and includes an explicit "inequality averseness" parameter that could allow inequality to be evaluated across a range of societal viewpoints. The downside of selecting a single indicator is that it provides only one statistical formulation and one combination of viewpoints from Tables 1 and 2. Using Theil's indices in addition to the Atkinson index can address the former concern, although the functional forms are quite similar and the measures have been highly correlated in past applications [61]. Simultaneously evaluating the extended Gini index could address the latter concern, but may only be interpretable in selected contexts (i.e., where subgroup decomposability is unimportant and where it is logical to consider ranks rather than relative values). In our illustrative example, we therefore focus on the Atkinson index but provide some sensitivity calculations using the Gini index and Theil's indices.

### Illustrative Example

To demonstrate the potential applicability of inequality indices for health benefits analysis, we provide an illustrative pollution control example. Suppose that there are 10 geographic areas affected by a given pollutant, numbered sequentially 1 through 10. Each area contains 100 people of either Type A or Type B, where Type B represents a high-risk population of concern to policymakers. Table 4 shows the number of people of each type in each area, as well as the initial exposure to some pollutant, which for simplicity corresponds to the area number. We assume that the risk from this pollutant is twice as high for Type B people than for Type A people. Since the selected inequality measures are scale invariant, for simplicity, we assign a risk per unit exposure of 2 for Type B people and 1 for Type A people. Thus, at baseline, we have a total risk of 8800 units, of which 6600 are associated with Type B and 2200 with Type A people. We consider this to be Scenario 1.

**Table 4:** Baseline Exposure and Population Characteristics for Illustrative Pollution Control Example (Scenario 1)

Geographic Area/Exposure Level	# Type A People	# Type B People	Individual Risk, Type A People	Individual Risk, Type B People	Total Risk, Type A People	Total Risk, Type B People
1	100	0	1	2	100	0
2	90	10	2	4	180	40
3	80	20	3	6	240	120
4	70	30	4	8	280	240
5	60	40	5	10	300	400
6	50	50	6	12	300	600
7	40	60	7	14	280	840
8	30	70	8	16	240	1120
9	20	80	9	18	180	1440
10	10	90	10	20	100	1800
Total	550	450			2200	6600

Now, suppose that we have the opportunity to remove 5 units of exposure by reducing exposure by 50% in any geographic areas we choose. Note that elimination of exposure is not considered (as this would cause errors in the Atkinson index for values of e greater than 1, and is usually unrealistic in a pollution control setting), and partial reductions are not considered for simplicity's sake. For each control strategy (consisting of 50% exposure reductions in a subset of geographic areas), we can therefore determine the total health benefits, the change in the Atkinson index for a given value of θ (or the change in other indices), the change in the between-group Atkinson (i.e., inequality between A and B), and the change in the within-group Atkinson (i.e., inequality across geographic areas).

First considering the baseline level of inequality, total inequality with the Atkinson index ranges from 0.14 for ε = 0.5 to 0.85 for ε = 10, demonstrating how increasing aversion to inequality raises the Atkinson index. The corresponding baseline inequality values are 0.41 for the Gini index, 0.35 for the mean log deviation, and 0.27 for Theil's entropy index. Note that little useful information can be gained by comparing the absolute magnitudes of these inequality indicators, other than the fact that the extent of inequality depends on the indicator and/or parameter. The key in this context is whether the policy decision would be sensitive to the choice of indicator.

**Figure 1:**
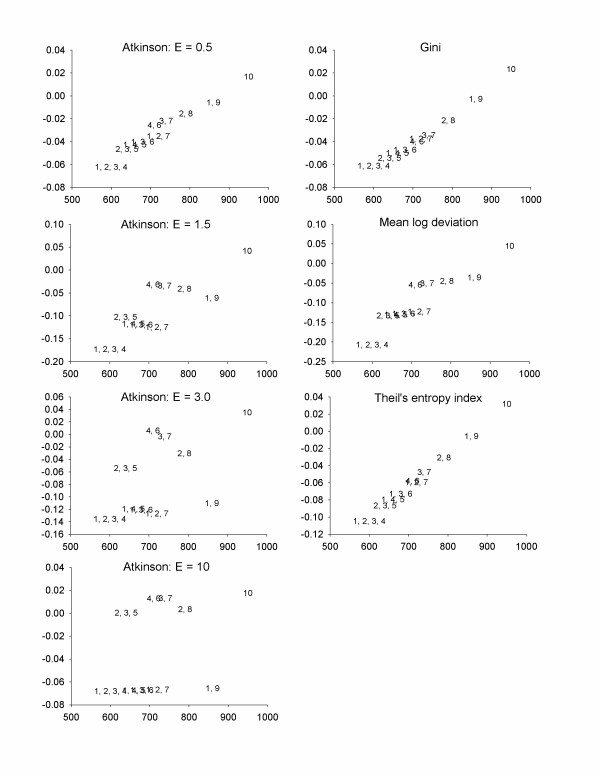
Health benefits and changes in inequality indicators for Scenario 1; High-risk people are highly exposed – see Table 4 for details. The numbers in the plot refer to the geographic areas in which exposure was reduced by 50%. X-axes = Health benefits, Y-axes = inequality benefits (positive numbers imply decreases in inequality).

**Figure 2:**
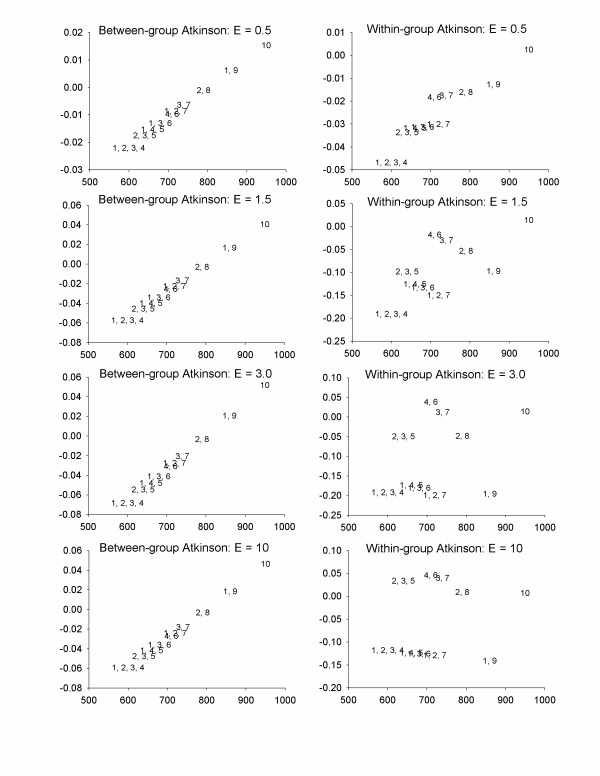
Health benefits and changes in between-group and within-group Atkinson index for Scenario 1; High-risk people are highly exposed – see Table 4 for details. The numbers in the plot refer to the geographic areas in which exposure was reduced by 50%. X-axes = Health benefits, Y-axes = inequality benefits (positive numbers imply decreases in inequality).

As shown in Figure 1, removing 5 units of exposure from Area 10 is optimal from both an efficiency and equality standpoint, based on the Atkinson index. This is unsurprising, as this reduces exposure from a site with the highest exposure and the greatest number of high-risk individuals. If for some reason this control strategy is not viable, there is no clear-cut optimal strategy. For small values of ε (representing less inequality averseness), the more efficient strategies are also preferable from an inequality perspective. As ε increases, we find an efficiency-equality tradeoff, with four control strategies considered viable for at least one value of ε (control at Areas 1 and 9, 2 and 8, 3 and 7, or 4 and 6). The remaining control strategies are strictly dominated, as they have both lower health benefits and a higher Atkinson index. Using the Gini index, the Theil' s entropy index, or the mean log deviation yields patterns similar to those for the Atkinson index with low values of ε, with more efficient strategies generally appearing more equal as well (Figure 1). Looking at between-group and within-group inequality (Figure 2) illustrates that the more efficient strategy is always preferable from a between-group inequality perspective, due to the fact that these strategies remove more exposure from larger groups of high-risk (Type B) individuals. The optimal strategies for within-group inequality are similar to that for total inequality.

Scenario 1 is fairly straightforward and yields uncontroversial findings, given that areas with more high-risk people also have higher exposures. Now, suppose that the situation is more complex (Scenario 2), where the geographic areas with more high-risk individuals have lower exposures (i.e., area 1 has an exposure of 10, and area 10 has an exposure of 1). For this scenario, all inequality indicators at baseline are lower (Atkinson of 0.07 for ε = 0.5 and 0.67 for ε = 10, Gini index of 0.28, mean log deviation of 0.24, Theil's entropy index of 0.13), reflecting a more equitable distribution of risks.

**Figure 3:**
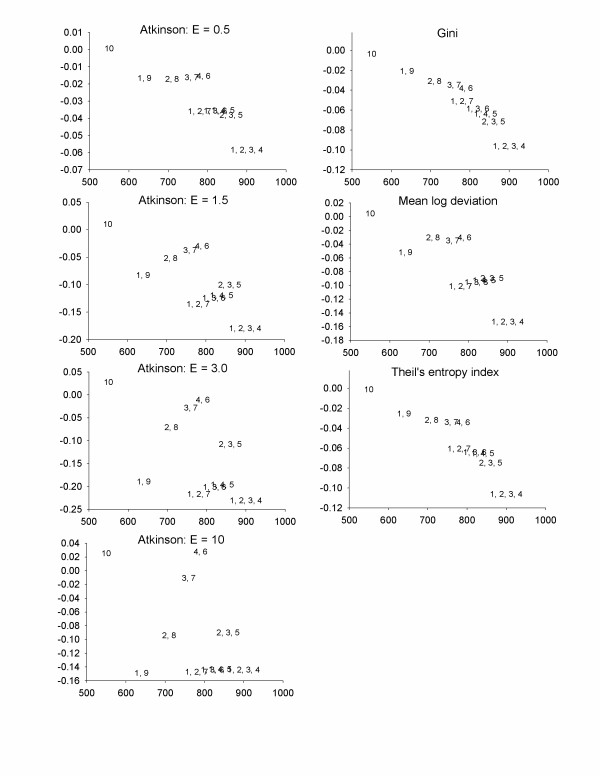
Health benefits and changes in inequality indicators for Scenario 2; Low-risk people are highly exposed – the scenario is as presented in Table 4, but area 1 has exposure 10, area 2 has exposure 9, etc. The numbers in the plot refer to the geographic areas in which exposure was reduced by 50%. X-axes = Health benefits, Y-axes = inequality benefits (positive numbers imply decreases in inequality).

Figure 3 illustrates that there is no single dominant policy strategy based on the Atkinson index, regardless of the value of ε used. Of the 10 policy options, six are strictly dominated from both efficiency and equality standpoints for all values of ε and would not be considered further. Choosing among the remaining policies depends on the degree to which one is willing to trade increases in efficiency for decreases in equality as well as on general preferences for equality. The Gini index, Theil's entropy index, and the mean log deviation yield the same four strategies on the optimal frontier as the Atkinson indices (controlling at 4 and 6; 2, 3, and 5; 1, 2, 3, and 4; and 10).

**Figure 4:**
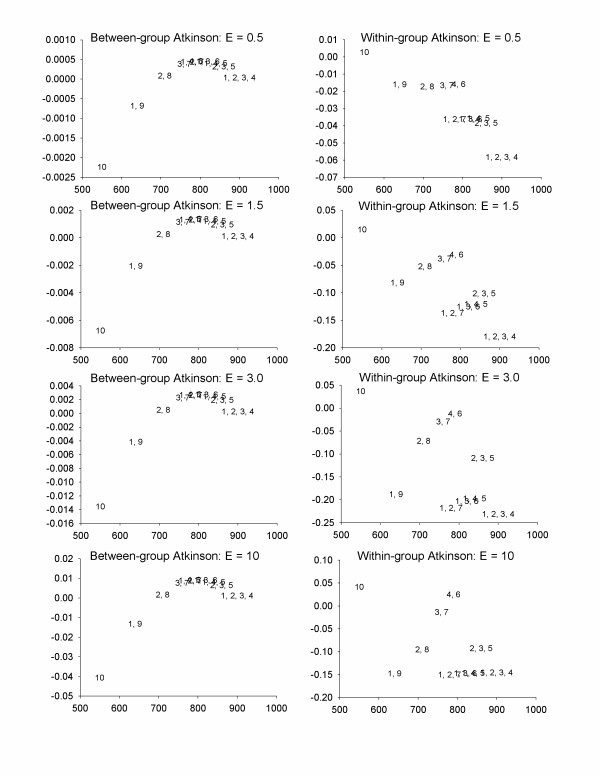
Health benefits and changes in between-group and within-group Atkinson index for Scenario 2; Low-risk people are highly exposed – the scenario is as presented in Table 4, but area 1 has exposure 10, area 2 has exposure 9, etc. The numbers in the plot refer to the geographic areas in which exposure was reduced by 50%. X-axes = Health benefits, Y-axes = inequality benefits (positive numbers imply decreases in inequality).

For Scenario 2, between-group inequality is lower than in the previous case (Figure 4). A subset of five policy options would reduce between-group inequality (although these are not identical to the optimal policy options for total inequality). Given small between-group inequality, within-group inequality is nearly identical to total inequality (Figure 4). If we wanted to restrict ourselves to policies that were in the optimal frontier from all three perspectives for all values of ε evaluated, we would choose between controlling at Areas 1, 2, 3, and 4 and controlling at Areas 2, 3, and 5.

## Discussion

We have demonstrated that it is plausible to quantify changes in inequality within a health benefits analysis, potentially informing concerns about inequity. The Atkinson index is the single indicator that best fulfills basic axioms and has two key characteristics crucial for health benefits analysis – the ability to evaluate the indicator for a variety of societal weights attached to inequality, and the ability to decompose the indicator to determine subgroup inequality. Additional indicators that have more limited applicability and interpretability, such as the Gini index, the Theil's entropy index, or the mean log deviation, allow us to evaluate the sensitivity of our findings to the underlying structure of the indicator. Our illustrative example demonstrates how efficiency-equality tradeoffs can be explicitly considered, at least in a simplified context, and how the subset of policy choices can be limited to those on the optimal frontier across levels of inequality averseness.

Although our analytical approach provides a mechanism to incorporate inequality and inequity issues into health benefits analysis, there are numerous limitations, and it is important to acknowledge that this sort of analysis will only be meaningful in selected contexts. First, the Atkinson index reflects only one way of measuring individual complaints and aggregating across those complaints: a "relative to the average" view (Table 1) combined with a "weighted additive" principle of equality (Table 2) [36]. As articulated above, environmental justice advocates may be more concerned with comparisons to all those better off, while health benefits analysis generally adopts an additive framework. Thus, the Atkinson index is a reasonable compromise between competing world views, but ultimately may not satisfy either one. Evaluating whether conclusions differ with the Gini index, which captures the above viewpoints but violates other criteria, can help to limit this issue.

In addition, we have only focused on the health benefits side of benefit-cost analysis and have not considered costs. Clearly, if the costs of an environmental regulation would be disproportionately borne by segments of the population, it would be important to incorporate that fact into the analysis. This adds another layer of analytical complexity, as the distributions of economic impacts of environmental policies are rarely estimated in enough detail to incorporate into our analytical framework. Future analyses should consider mechanisms to more explicitly incorporate the distribution of costs into our proposed framework.

Some of our proposed axioms also have implications that may not necessarily be desirable. For example, a scenario with uniform pollutant exposures and uniform relative risks from that pollutant could be considered "unequal" if baseline health disparities existed for other reasons. It could be argued that the equity-related goal of environmental policy should be to achieve uniformity in pollutant exposures across all individuals, with underlying health disparities to be resolved through other policy mechanisms. However, the Clean Air Act (42 U.S.C.A. 7408 as amended) requires the US Environmental Protection Agency to "protect the health of sensitive or susceptible individuals or groups". Thus, the EPA would consider lower pollution exposure necessary for individuals who are more sensitive to that pollutant, which implies that differential exposures would be desirable.

The above limitations are largely theoretical, but there is a significant practical barrier in implementing our proposed framework. Information about differential risk per unit exposure is rarely available to the extent necessary, especially stratified across sociodemographic covariates of interest [21]. This limitation can be clearly seen within recent regulatory impact analyses [48,49], which estimated health benefits assuming all subpopulations to have identical susceptibility and provided no spatial or demographic breakdowns of benefits. It is this historical omission within risk assessment of geographic or demographic patterns of risk, susceptible subpopulations, or cumulative environmental burdens that led many environmental justice advocates to question its applicability [18,79,80]. Even setting aside this problem, the welfare measure of concern in this case (unlike measures such as income) is clearly hard to quantify with precision. Uncertainty in the welfare measure would be important to explicitly incorporate into the analysis, as non-linear inequality indicators may lead to important differences between the inequality of the expected values of the welfare measures and the expected value of the inequality measure. These limitations do not preclude the development of a theoretical framework for incorporating equity into health benefits analysis, but they provide a cautionary note about implementation.

In addition, while our approach allows certain policy options to be rejected due to strict dominance across both efficiency and equality measures, it does not help decision-makers choose among policy options that lie on the optimal tradeoff frontier. Further research would be needed to ascertain the degree to which individuals are willing to trade off efficiency for equality, as well as the dependence of this tradeoff on the absolute level of risk and the policy context.

A final limitation is perhaps the most significant – in spite of some of the appealing aspects of our proposed framework, it is possible that neither risk assessors nor environmental justice advocates will consider these indicators meaningful enough to influence policy decisions. Risk assessors have raised concerns previously that narrowly focused conceptualizations of equity could produce perverse and undesirable outcomes [81] and that attempts to incorporate distributional concerns into an analysis are doomed to be both crude and controversial [82]. Similarly, our approach could be considered as reductionist and outcome-driven by environmental justice advocates, not addressing their core concerns.

However, it is important to recognize the current state of the science and the advancements our framework provides. The literature to date has been limited, and the few applications that considered methods to quantify the distribution of risk used the Gini coefficient without consideration of its underlying meaning [12,13,18], and did not apply the inequality indicator in an interpretable fashion. An axiomatic approach that allows for an array of conceptualizations of equity would start to respond to the concerns of risk assessors. Moreover, process equity is generally viewed as a means to achieve outcome equity, and perceptions about outcomes may influence perceptions about process, and vice versa [83]. Thus, our framework should contribute to better linkages between environmental justice and risk assessment applications, even if it cannot resolve the core philosophical differences between these disciplines.

## Conclusion

We have proposed an axiomatic approach to develop inequality indicators that are meaningful in health benefits analysis, an important application of risk assessment methodology. The Atkinson index best satisfies our major axioms, and if applied appropriately, can help to quantify the changes in equality of health risk at the same time as changes in the magnitude of health risk are estimated. An illustrative pollution control example demonstrates the efficiency-equality tradeoffs that may be present and the degree to which they depend on society's inequality averseness. Further study will focus on the application of the Atkinson index (and other meaningful inequality indicators) in actual health benefits analysis situations to determine the practical significance and the data limitations (i.e., evaluating the efficiency and equity implications of various power plant pollution control strategies). In addition, future studies should explore the equity perceptions of key stakeholders to allow for refinement of the analytical framework.

## List of abbreviations

SQV – Squared coefficient of variation

## Competing interests

The author(s) declare that they have no competing interests.

## Authors' contributions

JIL conceived of the study and was responsible for the axioms for inequality and the illustrative example, as well as the drafting of the manuscript. SMC evaluated the literature on previous inequality indicators. JLT developed the definitions and concepts of equality and equity. All authors contributed to the writing and reviewing of the manuscript and agree on its contents.
